# Use of biomarkers in pediatric sepsis: literature review

**DOI:** 10.5935/0103-507X.20160080

**Published:** 2016

**Authors:** Vanessa Soares Lanziotti, Pedro Póvoa, Márcio Soares, José Roberto Lapa e Silva, Arnaldo Prata Barbosa, Jorge Ibrain Figueira Salluh

**Affiliations:** 1 Instituto D'Or de Pesquisa e Ensino - Rio de Janeiro (RJ), Brazil.; 2 Universidade Federal do Rio de Janeiro - Rio de Janeiro (RJ), Brazil.; 3 NOVA Medical School, Universidade Nova de Lisboa - Lisboa, Portugal.; 4 Polyvalent Intensive Care Unit, Hospital de São Francisco Xavier, Centro Hospitalar de Lisboa Ocidental - Lisboa, Portugal.

**Keywords:** Sepsis, Biomakers, Child, Intensive care units, pediatric, Sepse, Biomarcadores, Criança, Unidades de terapia intensiva pediátrica

## Abstract

Despite advances in recent years, sepsis is still a leading cause of hospitalization and mortality in infants and children. The presence of biomarkers during the response to an infectious insult makes it possible to use such biomarkers in screening, diagnosis, prognosis (risk stratification), monitoring of therapeutic response, and rational use of antibiotics (for example, the determination of adequate treatment length). Studies of biomarkers in sepsis in children are still relatively scarce. This review addresses the use of biomarkers in sepsis in pediatric patients with emphasis on C-reactive protein, procalcitonin, interleukins 6, 8, and 18, human neutrophil gelatinase, and proadrenomedullin. Assessment of these biomarkers may be useful in the management of pediatric sepsis.

## Abstract (pt)

A despeito dos avanços nos últimos anos, a sepse ainda é uma das principais causas de internação e mortalidade em lactentes e crianças. A presença de biomarcadores na resposta a um insulto infeccioso resulta em seu uso na triagem, no diagnóstico, no prognóstico (estratificação de risco), na monitorização da resposta terapêutica e no uso racional de antibióticos (duração adequada, por exemplo). Os estudos sobre biomarcadores na sepse em crianças são ainda relativamente escassos. Esta revisão aborda o uso de biomarcadores na sepse em pacientes pediátricos, com ênfase em proteína C-reativa, procalcitonina, interleucinas 6, 8 e 18, gelatinase dos neutrófilos humanos e proadrenomedulina, que podem ser úteis na abordagem da sepse pediátrica.

## INTRODUCTION

Sepsis is a leading cause of hospitalization in pediatric intensive care units.^(1,2)^ In the last decade, a series of initiatives were implemented that aim not only to improve the understanding of sepsis and the clarity of concepts related to this condition^(3,4)^ but also to reduce morbidity and mortality due to sepsis through earlier diagnosis and initiation of antibiotic therapy as well as through the provision of specific guidelines for the treatment of pediatric sepsis.^(5)^ Despite these measures and the lower mortality from sepsis in children compared to adult patients, the impact of sepsis in the pediatric population remains high. According to the World Health Organization, sepsis remains a leading cause of death in infants and children in developed and developing countries.^(4)^

Recognizing the complexity of sepsis and the inadequate clinical concepts associated with the condition, a different conceptual approach based on a system similar to the tumor-node-metastasis (TNM) cancer staging system, the PIRO (acronym for Predisposition, Insult, Response and Organ Dysfunction) concept, was developed and proposed in the sepsis consensus published in 2003.^(6)^ Sepsis staging into these four domains allows stratification of its treatment by individualizing the treatment used in each domain.^(7)^ The host response to infection is known to be variable and individual, involving increased levels of biomarkers and biomediators that participate in the inflammatory response to the infectious insult. The specific response of any patient depends on the focus of infection, the pathogen causing the infection, and the host (genetic predisposition and coexisting diseases), and different responses occur at the local, regional, and systemic levels.

**Figure 1:**
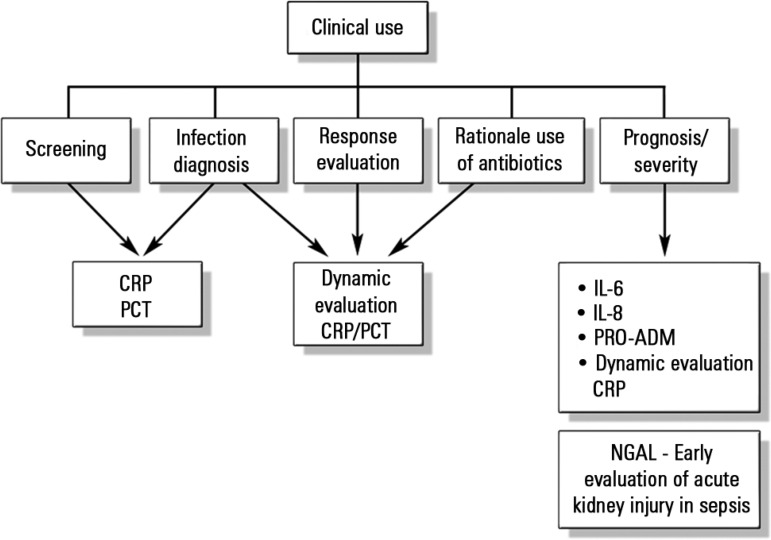
Biomarkers in pediatric sepsis. CRP - C-reactive protein; PCT - procalcitonin; IL-6 - interleukin 6; IL-8 - interleukin 8; NGAL - human neutrophil gelatinase.

The known presence of specific biomarkers during the response to an infectious insult makes possible the potential clinical use of such biomarkers in screening, diagnosis, prognosis (risk stratification), therapeutic response monitoring, and rational use of antibiotics (determination of adequate treatment length, for example) (Figure 1).

A biomarker^(8,9)^ may be defined as a characteristic that can be objectively measured and assessed as an indicator of normal biological processes, pathological processes, and/or pharmacological responses to a therapeutic intervention.

Evidences for/on the use of biomarkers have been studied and confirm that their use should be judicious, and their indications, use and applicability should be understood in association with clinical evaluation.^(10,11)^ This review article examines the use of biomarkers in pediatric patients with sepsis. A literature review was performed after searching for articles with the terms biomarkers AND children AND pediatric AND sepsis in the MEDLINE/PubMed database (http://www.pubmed.gov) published until May 1, 2016, without language restrictions. A total of 274 articles, including original and review articles, were found.

The use of biomarkers for screening and diagnosis has been studied for some time. Biomarkers for early detection of conditions with a worse prognosis and markers with improved correlation with clinical severity are already available, and their use as risk and prognostic stratifiers is promising. However, studies of the use of biomarkers in the pediatric age group are still very limited compared with studies performed in adults and even in the neonatal population. Some biomarkers (Table 1) have been more commonly studied in the pediatric age group, and those will be addressed here.

**Table 1:** Main biomarkers in pediatric sepsis

Biomarker	Why use?	Limitations
CRP	Easily available and low cost Peaks 36 - 50 hours after an inflammatory trigger Serial use for therapeutic response assessment Not affected by immunosuppression, renal dysfunction or corticosteroid use	Variable sensitivity and specificity for detecting bacterial infection (lower when a single measurement is performed) Low accuracy
PCT	Peaks 24 - 36 hours after an inflammatory trigger More specific for bacterial infection	Variable sensitivity and specificity Altered serum levels in cases of renal dysfunction Lack of multicenter and prognostic and risk stratification studies Higher cost
IL-6 and IL-8	Increased accuracy when combined with other biomarkers Good correlation with severity Promising use in pediatric patients with cancer and febrile neutropenia	Few studies in the pediatric population
Adrenomedullin (proADM)	Correlation with severity and potential use as a risk stratifier Promising marker of diagnosis of infection in febrile neutropenic patients	Studies in the pediatric population are still scarce Not yet available for use in clinical practice
NGAL	Promising biomarker of acute kidney injury (organ dysfunction) Early increase in cases of acute kidney failure (48 hours prior to the increase of creatinine) Early introduction of renal protection measures	Lacks validation in pediatric patients with septic shock (low specificity as a kidney injury predictor) Low availability for use in clinical practice

CRP - C-reactive protein; PCT - procalcitonin; IL-6 - interleukin 6; IL-8 - interleukin 8; NGAL - human neutrophil gelatinase.

### C-reactive protein

C-reactive protein (CRP), one of the biomarkers that has been in longer use in pediatric sepsis, is a non-specific, acute-phase protein that increases 4-6 hours after exposure to an inflammatory trigger (infectious or not) and has an 8-hour doubling time, peaking from 36 to 50 hours after the trigger stimulus. CRP has a 19-hour half-life. Its levels decrease rapidly with the resolution of inflammation and is usually high in invasive bacterial infections.^(12)^

Regarding to the use of CRP as a diagnostic biomarker, when considered in a single dosage, its sensitivity and specificity are limited for differentiating between severe bacterial infection and benign or non-bacterial infection, for example, in cases of pediatric emergency. In a systematic review of CRP diagnostic accuracy for bacterial infection in non-hospitalized children with fever, the sensitivity and specificity of CRP were estimated at 77% and 79%, respectively.^(13)^ However, its predictive value increases with the number of serial measurements, thus rendering it possibly useful for therapeutic management. Serial measurements in which CRP levels remain elevated or increase after 48 hours of antibiotic therapy suggest treatment failure.^(12)^

Studies in neonates and young infants indicate that increases in the levels of CRP of less than 10mg/L in samples collected at 24-hour intervals are useful for excluding the diagnosis of infection and/or suspected sepsis.^(12,14)^ This type of monitoring may permit the discontinuation of antibiotic therapy in selected patients and make it possible to avoid the use of antibiotics for an extended and unnecessary period of time.

A recent study of septic neonates showed that serial CRP measurements during the first 48 hours of antibiotic therapy may help predict whether the causative agent is sensitive to the antibiotic regimen used; thus, serial CRP measurement can be a good predictor of adequate empirical antibiotic therapy. The decrease in CRP in this period identified whether the organism was sensitive, with 89% sensitivity and 80% specificity.^(15)^

Due to the limited specificity of CRP, the combined use of CRP with other biomarkers has been tested.^(13)^ Studies in children with febrile neutropenia included CRP evaluation as a predictor of severe sepsis in these patients. When combined with another biomarker, including interleukin 8 (IL-8), increased CRP levels are apparently a good diagnostic predictor in the first 24 hours.^(16)^ However, the accuracy of CRP alone for the diagnosis of severe bacterial infection (sepsis/severe sepsis) in these patients with cancer and febrile neutropenia is lower than that of other biomarkers (including interleukin 6 [IL-6] and procalcitonin [PCT]).^(17)^

Studies in critically ill adults, especially in patients with severe community-acquired pneumonia, have shown that monitoring serial CRP values and their variation during the first 5 to 7 days of clinical evolution in response to antibiotic therapy has better value as a prognostic predictor than using only the absolute values.^(18-20)^ Considering that CRP measurements have already been extensively used in clinical practice for many years and that they can be obtained with easy access and low cost and are available in most healthcare facilities, CRP is confirmed as a key biomarker of response to antibiotic treatment when analyzed dynamically. This type of analysis thus deserves a similar evaluation in pediatric patients; such evaluation has already been reported, albeit preliminarily.^(21)^

It is worth remembering that CRP is not a specific biomarker for differentiating infection from inflammation or for identifying specific infectious agents. As in the case of other biomarkers, its use should always be associated with bedside clinical evaluation of patients, and other clinical decision-making criteria should always be used. When available, the use of CRP combined with other biomarkers including procalcitonin (PCT), IL-6 and IL-8 to increase its specificity in the diagnosis of infections^(16,17)^ and to assess changes in changes in therapeutic approaches, including changes in antibiotic therapy, is also promising.

Despite its low specificity, CRP has unique characteristics that are advantageous for its use in critically ill patients.^(22)^ These include the fact that CRP is apparently little affected by the use of systemic corticosteroids; if the cause of its elevation is infectious, its concentrations are not changed by immunosuppression (including in critically ill adult patients with sepsis and neutropenia, for example). Furthermore, in contrast to other biomarkers, including PCT, CRP levels are unaffected by renal dysfunction or dialysis techniques.^(22,23)^

Thus, although CRP has been one of the best-known inflammatory biomarkers for many decades, the dynamic and judicious use of CRP combined with clinical criteria and/or other biomarkers has great value and should be considered systematically in sepsis treatment evaluation (Table 2).

**Table 2:** Main publications on the use of C-reactive protein in pediatric infection/sepsis

Authors	Type of publication	Main results	Conclusion
McWilliam et al.^(12)^	Narrative review	Single measurement of CRP is not sensitive or specific enough to identify severe bacterial infection Increased CRP suggests severe bacterial infection and requires further investigation	Serial measurements of CRP are useful in assessing the response to antimicrobial treatment CRP values that fail to decrease or continue to rise after 48 hours of antibiotic therapy suggest treatment failure
Sanders et al.^(13)^	Systemic review	Diagnostic accuracy of CRP for bacterial infection in non-hospitalized children with fever has 77% sensitivity and 79% specificity Increased predictive value with serial measurements	CRP is useful (moderately and independently) for the diagnosis and exclusion of severe bacterial infection Moderate sensitivity (77%) means that CRP may not be used to exclude all bacterial infections Serial measurements are even more useful
Santolaya et al.^(16)^	Prospective cohort	447 episodes of high-risk febrile neutropenia - 17% with diagnosis of severe sepsis Combination of 3 factors (age > 12 years, CRP > 90mg/L and IL-8 > 300pg/mL) on admission and/or 24 hours later identified risk for severe sepsis (6.7 RR)	Validation of the predictive risk model for severe sepsis in patients with high-risk febrile neutropenia in the first 24 hours of admission Proposal to incorporate this model in the initial evaluation of patients and more selective management of children at risk for severe sepsis
Kitanovski et al.^(17)^	Prospective cohort - 47 children	18 of 90 episodes of febrile neutropenia were classified as bacteremia/sepsis At days 1 and 2, CRP and other biomarkers had low to moderate diagnostic accuracy for sepsis with no significant difference between biomarkers The diagnostic accuracy of CRP was lower than that of IL-6 and PCT in severe sepsis	On admission and 24 hours later, the diagnostic accuracy of CRP alone for severe sepsis in children with febrile neutropenia was lower than that of PCT and IL-6
Lanziotti et al.^(21)^	Prospective cohort (preliminary results) - 57 children	50 of 57 patients were classified according to a CRP response pattern in the first week of antibiotic therapy in pediatric sepsis Mortality in the pediatric ICU was significantly different according to the CRP response pattern. Patients with decreased CRP had lower mortality than those without a decrease in CRP	Sequential CRP assessment (CRP ratio) is useful in the early identification of patients with poor prognosis Evaluation of CRP response in the first 7 days of antibiotic treatment may be useful in identifying an individual clinical course, influencing bedside decision-making

CRP - C-reactive protein; IL-8 - interleukin-8; RR - relative risk; IL-6 - interleukin-6; PCT - procalcitonin; ICU - intensive care unit.

### Procalcitonin

PCT, precursor of the calcitonin hormone, is secreted in healthy patients by neuroendocrine C cells of the thyroid, with minimal serum levels in these situations. However, during systemic infection, PCT is secreted by several other tissues, resulting in a considerable increase in serum levels. For this reason, PCT has been considered a reliable biomarker for differentiating sepsis from non-infectious systemic inflammatory response syndrome (SIRS). PCT may be useful for determining whether the use of antibiotics is required^(24)^ because it is attenuated by interferon gamma (IFN-γ) during viral infection and its level is related to the presence of bacterial infection. Considering the maximum sensitivity and specificity of the assay, PCT values lower than 0.5ng/mL are suggestive of inflammation without infectious etiology, and values higher than 2.0ng/mL are suggestive of sepsis.^(25)^ However, other studies in adults^(26)^ have used values different from these, considering PCT values higher than 0.25 to 0.5ng/mL as reflecting probable bacterial infection and indicative of required antibiotic therapy. It is important to remember that these values have also varied with advances in PCT detection methods.

Elevation of PCT levels usually occurs earlier during the course of infection than elevation of CRP levels, peaking at approximately 24 - 36 hours. Some studies of critically ill pediatric patients^(27,28)^ showed that the accuracy of PCT measurement in detecting bacterial infections is better than that of other markers, especially CRP. However, its sensitivity and specificity vary.

A meta-analysis published in 2014 on the use of PCT levels for evaluating febrile infants (7 studies involving 2,317 patients) for severe bacterial infection showed that serum values lower than 0.3ng/dL may be useful for excluding severe infection when used as complementary to clinical evaluation, which should remain the key factor for deciding the therapeutic approach in these patients.^(29)^

Clinical judgment is still the most valuable tool for defining the initiation of antibiotic therapy in patients. However, over the course of many infectious diseases, clinical evaluation becomes more nonspecific and sometimes occurs too late to provide a basis for decision-making regarding antimicrobial treatment.^(23)^ In this context, the use of biomarkers plays a key role, and the use of PCT has been extensively studied in this regard. Various studies have shown that serial PCT measurements may be a good indicator of when the use of antibiotics may be safely suspended in pediatric patients with sepsis.^(28,30)^ Thus, the use of PCT may make it possible to reduce the duration of antimicrobial treatment and may contribute to reducing bacterial resistance to antibiotics and to minimizing the adverse effects of these drugs, including nephrotoxicity and ototoxicity. In adult patients, studies of the use of PCT to guide antibiotic therapy discontinuation are more numerous, albeit limited even in this population;^(23)^ these studies feature heterogeneous PCT use protocols, high rates of exclusion of patients, long antibiotic therapy in control group patients, and lack of further prognostic information, including length of hospital stay and mortality.

Studies on serial PCT measurements at patient admission and throughout hospitalization have been performed to correlate this biomarker with disease severity, multiple organ failure, and mortality.^(27,28,30,31,32)^ These studies indicate that serial PCT measurements are considered a possible marker of prognosis.

In a cohort in an American tertiary hospital (78 children with criteria for sepsis and septic shock and 12 critically ill children without sepsis), persistently high PCT in children with bacterial sepsis was related to a poor outcome.^(31)^ In another prospective observational study in a pediatric intensive care unit in São Paulo, Brazil, involving 689 patients admitted within 2 years and including 59 children with criteria for sepsis and 65 children with criteria for septic shock, the plasma levels of PCT at admission allowed differentiation between sepsis and septic shock.^(33)^ These results suggest the validity of using PCT in auxiliary diagnosis of septic conditions in children and its potential usefulness as an indicator of disease severity; the latter may be useful for evaluating the appropriateness of patient hospitalization in the pediatric intensive care unit, for example.

A cohort study published in 2015 of 82 children diagnosed with meningitis demonstrated that serum PCT levels are related to the severity of infection in patients with bacterial meningitis and that a decrease in PCT levels in response to treatment was a good predictor of favorable prognosis.^(34)^ A meta-analysis published in the same year showed that PCT is highly accurate in differentiating bacterial and viral meningitis in children, with 96% sensitivity and 89% specificity.^(35)^

Another meta-analysis published in 2015 involving the use of PCT for the diagnosis of acute pyelonephritis in pediatric patients showed that PCT values greater than or equal to 1.0ng/mL had better diagnostic performance (91% specificity) than values greater than or equal to 0.5ng/mL (76% specificity and 86% sensitivity).^(36)^

PCT has also been used as an indicator of sepsis and bacteremia in children with cancer and febrile neutropenia, and its accuracy in some studies is better than that of CRP. PCT values are apparently unaffected by the use of chemotherapy and corticosteroids, and its use in the stratification of cancer patients with febrile neutropenia has been encouraged in recent years.^(22,37,38)^

Although still few in number, studies are being conducted on the use of PCT in situations requiring the differentiation of SIRS from sepsis, including in patients in cardiac surgery post-operative care,^(39,40)^ wherein the use of antibiotics may aggravate nephrotoxicity in patients subjected to intraoperative extracorporeal circulation. The use of PCT testing in severe burn victims has also been assessed, although its use as a predictor of infection or mortality in burned children is still limited.^(41,42)^

Limited evidence of the use of PCT as a prognostic predictor in children has been published. A recent single-center prospective cohort study of 62 children diagnosed with SIRS and sepsis showed that higher PCT values were found in patients with Pediatric Logistic Organ Dysfunction (PELOD) scores greater than or equal to 12 than in patients with scores lower than 12 during the first 5 days of hospitalization. This study showed that PCT levels were related to severity of infection and to organ dysfunction in patients with sepsis, although they did not show a relationship to mortality.^(43)^

The use of PCT in risk stratification and prognosis and/or mortality prediction has been increasingly evaluated, although more robust studies in pediatric patients are needed. The existing studies are mostly single-center and include a small number of patients. The levels of evidence published to date (Table 3) still preclude considering PCT a biomarker for routine use in clinical practice as a risk stratifier and a prognostic predictor or even to guide the duration of antibiotic treatment and bedside decision-making. Multicenter studies involving higher numbers of patients, preferably in different regions of the world, must be performed. It is important to remember that the cost of PCT testing is still relatively high and that Kryptor, the only method available for its measurement, is unavailable in many health facilities, especially in developing countries.

**Table 3:** Main publications of the use of procalcitonin in pediatric infection/sepsis

Authors	Type of publication	Methods and main results	Conclusion
Rey et al.^(27)^	Observational prospective cohort	359 patient-days included in the study Evaluation of the use of PCT, CRP and leukocyte count to classify: absence of infection, SRIS, localized infection, sepsis, severe sepsis and septic shock Area under the receiver operating characteristic (ROC) curve for diagnosis of sepsis was 0.532 for leukocyte count, 0.75 for CRP and 0.912 for PCT	PCT is a better diagnostic marker for sepsis in critically ill patients than CRP PCT and CRP may be useful as clinical tools to stratify the severity of patients with SRIS
Fioretto et al.^(28)^	Prospective cohort	87 patients (46 patients diagnosed with sepsis and 41 patients diagnosed with septic shock) PCT and CRP measurement on admission and 12 hours later	PCT was better than CRP for the diagnosis of sepsis and septic shock, particularly on admission, and was related to disease severity
England et al.^(29)^	Systematic review	7 studies of 2,317 patients Evaluation of the use of PCT in the diagnosis of severe bacterial infection in young infants (≤3 months of age) 5 of 7 studies used the same cut-off value (0.3ng/mL) RR for diagnosis of severe bacterial infection with increased PCT was 3.97 RR for diagnosis of severe bacterial infection using clinical prediction was 30.6 (patients without antibiotic treatment) and 8.75 (patients using antibiotic)	PCT values < 0.3ng/mL may be useful in the exclusion of severe bacterial infection, as an additional test to clinical prediction, remaining as a key factor to guide the therapeutic approach in these patients
Arkader et al.^(30)^	Observational prospective cohort	PCT and CRP kinetics studied in patients undergoing heart surgery with cardiopulmonary bypass (Group 1 - SRIS) and in patients with confirmed bacterial sepsis (Group 2) The area under the ROC curve was 0.99 for PCT and 0.54 for CRP	PCT was able to differentiate patients with SRIS and sepsis, and CRP was not PCT concentrations varied according to the progression of sepsis
Han et al.^(31)^	Prospective cohort	87 patients with sepsis and septic shock 12 critically ill patients with no criteria for sepsis PCT values were elevated in patients with bacterial sepsis at days 1 and 3 of pediatric ICU admission Persistently elevated PCT values were found in patients with bacterial sepsis with persistent multiple organ failure and in those who died, but not in patients with non-bacterial sepsis (fungal, viral or sepsis with negative culture)	PCT is persistently elevated in children with bacterial sepsis and poor prognosis
Hu et al.^(34)^	Prospective cohort	Investigation of the relationship between the PCT SL and prognosis in children with bacterial meningitis 82 patients included Patients with bacterial meningitis have higher PCT SL than those with viral meningitis PCT SL were significantly higher in patients with severe sepsis and septic shock than in patients with non-severe sepsis and without sepsis A drop in PCT SL was observed in patients with a good response to antibiotic treatment PCT SL were significantly higher in patients who died than in survivors	PCT SL are related to disease severity in children with bacterial meningitis A decrease in PCT SL after treatment may indicate a favorable prognosis
Henry et al.^(35)^	Systematic review	8 studies were included (616 patients) PCT SL were highly accurate in differentiating the diagnostic etiology of meningitis in children, with 96% sensitivity and 89% specificity In 6 studies, the accuracy of PCT was higher than that of CRP	PCT SL are highly accurate in differentiating bacterial meningitis from viral meningitis in children
Hatzistilianou et al.^(37)^	Prospective cohort	Assessment of PCT, CRP, TNF-alpha, IL-1b, IL-8 and TNF-receptor II values in the rapid and early diagnosis of infection in patients with acute lymphocytic leukemia and febrile neutropenia and differentiation between bacterial and viral infection The SL of biomarkers were assessed on admission and for 7 consecutive days PCT SL were significantly different between bacterial and non-bacterial episodes, with 94% sensitivity and 96.5% specificity	Serial PCT measurements may be useful in predicting severe sepsis in patients with acute lymphoid leukemia and febrile neutropenia
Zurek et al.^(43)^	Prospective cohort	62 patients (0 - 19 years) with SRIS or sepsis were included Severity measured using the PELOD PCT SL were measured from day 1 to day 5 of admission and significantly higher PCT values were found in patients with PELOD >12 than with PELOD < 12	PCT SL from day 1 to day 5 of pediatric ICU admission are related to severity and multiple organ dysfunction in children with SRIS/sepsis

PCT - procalcitonin; CRP - C-reactive protein; SRIS - systemic inflammatory response syndrome; RR - relative risk; SL - serum levels; TNF-alpha - Tumor necrosis factor alpha; IL-1b-interleukin 1 beta; IL-8 interleukin 8; TNF-receptor II - tumor necrosis factor receptor II; PELOD - Pediatric Logistic Organ Dysfunction score.

### Interleukin 6

Interleukin 6 (IL-6) is a pro-inflammatory cytokine that has been studied for many years in adults as a biomarker of sepsis. However, few studies have been conducted in pediatric patients. IL-6 serum levels are higher in children diagnosed with sepsis than in patients with noninfectious systemic inflammation only,^(43,44)^ and its diagnostic accuracy increases when combined with other diagnostic biomarkers, including CRP, for example.^(43)^ Furthermore, among children with sepsis, an increase in IL-6 level is associated with more severe cases,^(45)^ and its use in clinical practice can provide a good predictor of severe sepsis.

IL-6 has also been studied in children diagnosed with cancer and febrile neutropenia; it is a highly accurate diagnostic marker of bacteremia and clinical sepsis in these patients.^(16,17)^

However, the use of IL-6 testing in clinical practice is still limited not only because of its low availability and high cost but also because of the lack of robust studies justifying its use, especially in the pediatric population.

### Interleukin 8

Interleukin-8 (IL-8) is a pro-inflammatory cytokine that may predict the survival of critically ill children. IL-8 is responsible for chemotaxis and neutrophil activation and may be used as a risk stratification biomarker. In a genomic expression study in pediatric patients with septic shock,^(46)^ higher IL-8 levels were observed in children with septic shock who died than in survivors based on 28-day mortality data. The same first author published^(47)^ a study showing that IL-8 serum levels lower than or equal to 220pg/mL (measured in the first 24 hours of hospitalization) may predict the survival of children with septic shock at 95% probability. Thus, IL-8 could be used to exclude low-risk patients from intervention clinical trials.

IL-8 is also a potential risk stratifier in pediatric cancer patients with febrile neutropenia. A recent prospective cohort study showed that low IL-8 serum levels predicted a low risk of bacteremia, with 90% sensitivity and 98% negative predictive value. Further studies are needed to confirm these data.^(48)^ Similarly, IL-8 levels higher than 300pg/mL associated with increased CRP levels and age older than 12 years were related to a higher risk of severity in pediatric patients with cancer and febrile neutropenia.^(49)^

Conversely, a study of adult patients showed that in this population, IL-8 is apparently not a good biomarker of stratification, indicating that further studies including this age group should be conducted.^(50)^

### Interleukin 18

Interleukin-18 (IL-18), a pro-inflammatory cytokine produced by activated macrophages, participates in the induction of cellular immunity. Elevated IL-18 levels are found in inflammatory disease, including rheumatoid arthritis, neonatal infections, and sepsis.^(51-53)^ Studies of adult populations have shown that elevated concentrations of IL-18 are associated with poor prognosis in septic patients.^(53)^ Although IL-18 is a potential diagnostic and risk stratification biomarker, very few studies of its use as such a marker have been published, especially in the pediatric population, and further studies must be conducted in this area.

The aforementioned interleukins vary greatly in their serum concentrations, and assessment of their levels is still not used routinely in most adult and pediatric intensive care units; such use is virtually limited to research. Performing further studies, especially multicenter studies on the use of interleukins as biomarkers in pediatric sepsis, is crucial to better understand their usefulness and the possibility of using them in clinical practice. IL-6 and IL-8 are promising, especially in the stratification of pediatric patients with febrile neutropenia, a potentially more severe condition than that of a previously healthy patient with a severe bacterial infection.

### Human neutrophil gelatinase

Human neutrophil gelatinase (serum neutrophil gelatinase-associated lipocalin, NGAL) is a promising biomarker of acute kidney injury. Urinary NGAL was validated as an early biomarker of acute kidney injury in a prospective cohort study^(54)^ involving 140 children from 1 month to 21 years of age in which acute kidney failure was graded using the pRIFLE (Pediatric modified Risk Injury, Failure, Loss, End-stage Kidney Disease) criteria. In this study, the concentration of urinary NGAL increased as the pRIFLE score worsened in acute kidney injury two days before the increase in serum creatinine levels. That study presents a new possibility for early diagnosis and prevention of acute renal injury in critically ill pediatric patients.^(55)^

Conversely, serum NGAL has not yet been validated as a biomarker of acute kidney injury in pediatric patients with septic shock. Wheeler et al. showed that serum NGAL is highly sensitive, albeit non-specific, as a predictor of acute kidney injury in these patients, requiring further studies.^(56)^

An observational cohort study performed in critically ill children showed that urinary NGAL is unaffected by sepsis, supporting its role as a predictor of acute kidney injury. However, in patients with sepsis, serum NGAL alone is unable to differentiate patients with acute kidney injury from those without it.^(57)^

In the adult population, a recent study conducted in patients with sepsis showed that plasma NGAL apparently has high sensitivity as a diagnostic predictor of acute kidney injury.^(58)^

As promising early biomarkers of acute kidney injury, urinary (already validated as an early predictor of acute kidney injury) and serum (still considered nonspecific in this prediction) NGAL could, in clinical practice, determine the earlier introduction of renal protective measures, including the replacement of nephrotoxic antibiotics, initiation of water restriction, and even the initiation of hemodialysis or hemofiltration, thereby contributing to improved prognosis in critically ill patients with sepsis and renal dysfunction. Studies on the use of NGAL as a biomarker in these patients are still scarce, and more robust studies must be performed.

### Adrenomedullin (proadrenomedullin)

Adrenomedullin (ADM), a peptide produced by various tissues during physiological stress, has anti-inflammatory, antimicrobial, and vasoregulatory activities. Although promising, this innovative biomarker is rapidly metabolized in the circulation, complicating its measurement. Thus, its precursor, proadrenomedullin (proADM or the similar midregional-proADM [MR-proADM]), has received more attention as a biomarker because it is more stable and easier to measure. The increase in ADM in sepsis is explained by two mechanisms: (1) ADM synthesis increases during severe infections because it is a peptide related to the calcitonin gene; and (2) bacterial endotoxins and pro-inflammatory cytokines lead to increased ADM gene expression in several tissues.^(59)^ Furthermore, decreased renal metabolism may partly account for the increased serum levels of proADM during infection.^(59,60)^ An observational study performed in 95 pediatric patients with sepsis showed that the serum levels of MR-proADM in septic patients requiring mechanical ventilation and inotropes were significantly increased. This biomarker was correlated with severity and could be used as a risk and prognostic stratifier with a higher positive predictive value for prognosis (in-hospital mortality) than PCT and CRP.^(61)^

Recently, the use of ADM as a marker of infection was assessed in cancer patients with symptoms of febrile neutropenia. The serum levels of ADM in febrile neutropenic patients with microbiologically documented infection were higher than the levels in patients with clinical infection only or with fever of undetermined origin. ADM showed a stronger correlation as severity predictor than the other two tested biomarkers, CRP and PCT.^(62)^ In adults, ADM has been extensively studied not only in sepsis but also in specific infections such as severe community-acquired pneumonia.^(63,64)^

New studies in children are needed to evaluate the performance of ADM as a biomarker of sepsis and general infection to establish more clearly its role not only as a diagnostic marker but also as a risk and prognostic stratifier in clinical practice.

### Use of biomarkers in risk stratification in pediatric sepsis

The use of biomarkers in risk stratification of pediatric patients with sepsis is promising, albeit challenging. Thus far, no single biomarker alone can be used to predict with full certainty the specific outcome for each patient. Considering the complex immune response of each host and the genetic diversity of populations, it is highly unlikely that any single biomarker could be used to identify and stratify all pediatric patients with sepsis.

A risk stratification strategy involving multiple biomarkers may be required. Multiple biomarker models have already been used in adults. Wong et al. published a multibiomarker model for risk stratification of pediatric septic shock, the Pediatric Sepsis Biomarker Risk Model (PERSEVERE). Twelve biomarkers were previously chosen and measured in 220 children in the United States in the first 24 hours after hospital admission.^(65)^ Based on the results, a risk model for estimating mortality in children with septic shock using five biomarkers (C-C chemokine ligand 3 [CCL3], IL- 8, heat shock protein 70 kDa 1B [HSPA1B], granzyme B [GZMB], and matrix metallopeptidase 8 [MMP8]) was created and validated. This model has potential application to patient stratification and selection for clinical trials (for excluding and including patients with low and high risk of death, respectively), individual decision making and efforts to improve the quality of septic shock treatment.^(66)^ The PERSEVERE model was validated with a multicenter cohort from various pediatric intensive care units in the United States that included 182 children with septic shock. The five biomarkers were tested in these patients within 24 hours of the clinical onset of septic shock, and the accuracy of the multiple biomarker model for 28-day mortality risk in these patients was tested using statistical tests. The study cohort mortality was 13.3%, compatible with the percentages reported in the literature on pediatric sepsis mortality. The sensitivity and specificity of PERSEVERE in predicting mortality were 83% and 75%, respectively, with a 34% positive predictive value, albeit with a 97% negative predictive value.^(66)^ This study demonstrates the possibility of predicting the outcome in pediatric patients using biomarkers and supports their prognostic value. However, it is still unclear whether biomarker use can effectively guide treatment and modify the outcomes of pediatric patients with septic shock.

A recent study showed that using a panel of biomarkers consisting of angiopoietin-1, angiopoietin-2, and bicarbonate was a better predictor of severity in pediatric septic patients than the separate use of these biomarkers.^(67)^

The concomitant use of multiple biomarkers for risk stratification in pediatric sepsis patients is promising for improved severity stratification and mortality estimation in these patients and for improved patient selection for inclusion in clinical trials. These promising results should facilitate further studies in this age group of patients.

## CONCLUSION

The use of biomarkers in pediatric sepsis is promising, although biomarker use should always be correlated with clinical evaluation. The combined use of multiple biomarkers may increase the sensitivity and specificity of sepsis diagnosis and prognosis compared with the use of a single biomarker. Biomarkers such as C-reactive protein and procalcitonin have shown a key role in clinical practice - C-reactive protein, especially, for the evaluation of the response to the antibiotic treatment, when evaluated dynamically. Measurement of procalcitonin levels can guide the initiation or discontinuation of antibiotic therapy in patients with severe clinical infection, although its limitations, including false negatives, the effects of renal dysfunction on serum levels, and clinical studies with a broad exclusion of patients.
